# Integrative genomics identifies candidate microRNAs for pathogenesis of experimental biliary atresia

**DOI:** 10.1186/1752-0509-7-104

**Published:** 2013-10-20

**Authors:** Kazuhiko Bessho, Kumar Shanmukhappa, Rachel Sheridan, Pranavkumar Shivakumar, Reena Mourya, Stephanie Walters, Vivek Kaimal, Eric Dilbone, Anil G Jegga, Jorge A Bezerra

**Affiliations:** 1 Cincinnati Children’s Hospital Medical Center and Departments of Pediatrics, University of Cincinnati College of Medicine, Cincinnati, OH, USA; 2 Regulus Therapeutics, San Diego, CA, USA; 3 Enercon Services, Kennesaw, GA, USA

**Keywords:** Cholestasis, Extrahepatic bile duct, Informatics, Cholangiocytes

## Abstract

**Background:**

Biliary atresia is a fibroinflammatory obstruction of extrahepatic bile duct that leads to end-stage liver disease in children. Despite advances in understanding the pathogenesis of biliary atresia, very little is known about the role of microRNAs (miRNAs) in onset and progression of the disease. In this study, we aimed to investigate the entire biliary transcriptome to identify miRNAs with potential role in the pathogenesis of bile duct obstruction.

**Results:**

By profiling the expression levels of miRNA in extrahepatic bile ducts and gallbladder (EHBDs) from a murine model of biliary atresia, we identified 14 miRNAs whose expression was suppressed at the times of duct obstruction and atresia (≥2 fold suppression, P < 0.05, FDR 5%). Next, we obtained 2,216 putative target genes of the 14 miRNAs using in silico target prediction algorithms. By integrating this result with a genome-wide gene expression analysis of the same tissue (≥2 fold increase, P < 0.05, FDR 5%), we identified 26 potential target genes with coordinate expression by the 14 miRNAs. Functional analysis of these target genes revealed a significant relevance of miR-30b/c, -133a/b, -195, -200a, -320 and −365 based on increases in expression of at least 3 target genes in the same tissue and 1^st^-to-3^rd^ tier links with genes and gene-groups regulating organogenesis and immune response. These miRNAs showed higher expression in EHBDs above livers, a unique expression in cholangiocytes and the subepithelial compartment, and were downregulated in a cholangiocyte cell line after RRV infection.

**Conclusions:**

Integrative genomics reveals functional relevance of miR-30b/c, -133a/b, -195, -200a, -320 and −365. The coordinate expression of miRNAs and target genes in a temporal-spatial fashion suggests a regulatory role of these miRNAs in pathogenesis of experimental biliary atresia.

## Background

Biliary atresia, one of the most common causes of neonatal cholestasis, results from a fibro-inflammatory obstruction of extrahepatic bile ducts of unknown etiology. If diagnosed early, surgical intervention with hepatoportoenterostomy has the potential to restore bile flow, but ongoing liver injury results in rapid progression to cirrhosis and the need for liver transplantation by 2 years of age in ~50% of children [1]. Contemporary studies suggest that the pathogenesis of bile duct injury results from an interplay between environmental factors and a biological response that involves cells of the immune system, injury and proliferation of the biliary epithelium, and excessive deposition of extracellular matrix [2,3]. Insight into the activation of these processes was obtained from large-scale gene expression analyses in livers of infants at the time of diagnosis [4] and from extrahepatic bile ducts and gallbladder (EHBDs) obtained from neonatal mice in a well-established model of rotavirus-induced experimental biliary atresia [5]. In this model, Rhesus rotavirus type-A (RRV) infection within 2–3 days of life leads to an inflammatory obstruction of extrahepatic bile ducts within a week, with age-restricted susceptibility [6,7]. This model has been shown to recapitulate many features of the tissue injury and pathogenesis observed in humans with biliary atresia [2,8]. More recently, an investigation of livers from this mouse model suggested a potential role for microRNAs in pathogenesis of disease [9].

microRNAs (miRNAs) are short non-coding RNAs that regulate gene expression at post-transcriptional levels by facilitating degradation and/or inhibiting translation of target mRNAs. miRNAs may play important roles in the development of the hepatobiliary system,[10] and cell- and tissue-specific miRNA expression profiles are altered in some diseases, such as cirrhosis and cancers [11]. To obtain insight into the role of miRNAs in pathogenesis of biliary atresia, we performed comprehensive expression analyses of miRNAs and mRNA from EHBDs at the times of epithelial injury, lumenal obstruction, and atresia in the experimental mouse model. Using integrative in silico approaches for miRNA and mRNA expression profiles, we found a coordinated temporal expression of a selected number of miRNAs and their target genes, with the activation of molecular networks regulating organogenesis and immune responses. Spatially, the expression of these miRNAs was higher in EHBDs (relative to livers), and was localized in cholangiocytes and in stromal cells of the subepithelial compartment.

## Results

### Time-dependent suppression of miRNAs in EHBDs

To search for miRNAs with potential roles in the pathogenesis of biliary atresia, we first performed a comprehensive survey of miRNA expression patterns in extrahepatic bile ducts and gallbladder (collectively termed EHBDs) of neonatal mice challenged with RRV within 24 hours of birth. All datasets are deposited in the Gene Expression Omnibus repository (http://www.ncbi.nlm.nih.gov/geo/), with accession code GSE41595. We focused on the analysis of EHBDs, rather than the liver, because they are the primary site of tissue injury, beginning with an epithelial insult (3 days), followed by lumenal obstruction with inflammatory cells (7 days) and later by atresia (14 days) [5]. In these studies, all mice developed jaundice by 7 days, which persisted and became associated with poor weight gain until the time of death by 14 days after RRV challenge, as described previously [8,12]. Among a total of 578 miRNAs, 143 changed their expression levels in EHBDs after RRV challenge by at least 2 fold when compared to saline-injected control mice (P < 0.05, FDR 5%; Additional files 1, 2 and 3). Among these miRNAs, only miR-let-7e* was differentially expressed in the RRV group at early phases of epithelial injury, with a 25.8 fold decrease below controls (3 days), while a greater number of miRNAs was downregulated at 7 days (N = 133) and 14 days (N = 17). Interestingly, only 6 miRNAs were upregulated by more than 2-fold, which were all at day 7 after RRV challenge and included miR-29b, which was reported to be elevated in the liver of newborn mice subjected to the same experimental model (Additional file 4) [9].

**Figure 1:**
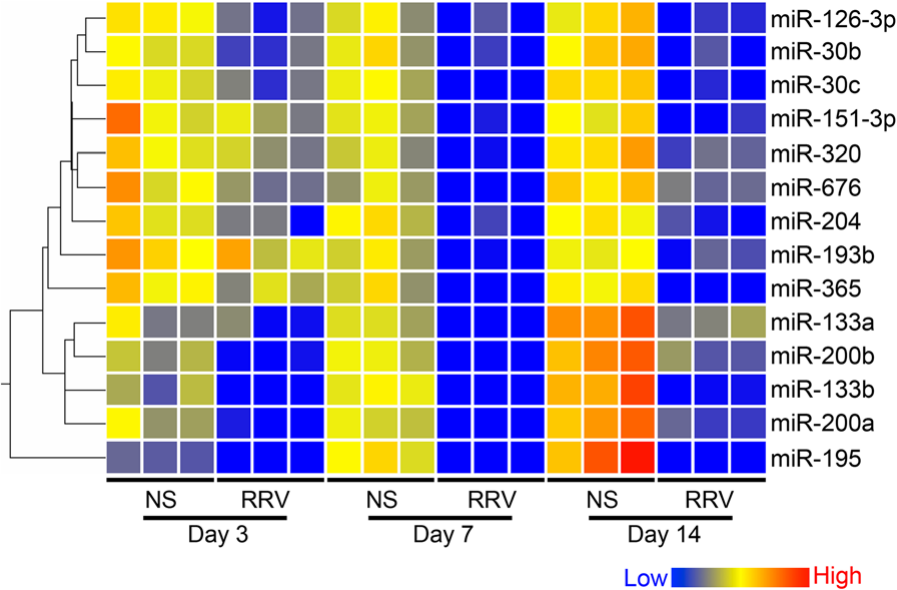
**Selection of miRNAs based on decreased expression at days 7 and 14 after RRV challenge.** One-way cluster analysis depicts the changes in expression levels of 14 miRNAs in EHBDs at 3, 7 and 14 days after RRV challenge relative to normal saline (NS) controls. Each column represents expression levels from pooled samples of 2–6 EHBDs. Expression levels are depicted as color variation from low (blue) to high (red).

To identify candidate miRNAs with a potential role in the pathogenesis of biliary injury, we selected miRNAs that showed the same pattern of expression at the times of bile duct obstruction and atresia (7 and 14 days, respectively). We found 14 miRNAs that were consistently suppressed at both time points (Figure 1 and Table 1); there was no miRNA that was overexpressed at both time points. Most of these miRNAs have been implicated in mechanisms of hepatocellular carcinoma (miR-193b, [13] -195, [14-16] -200a/b [15,17-19]), hepatitis C virus infection of hepatocytes (miR-193b [20] and −320 [21]), and nonalcoholic fatty liver disease and steatohepatitis (miR-200a/b, [22,23] Table 2). Most notably, miR-30b/c has been reported to influence transforming growth factor beta 1-induced epithelial to mesenchymal transition and biliary development and infection, [10,24-26] while miR-200b, [27] -204, [28] and −320 [28] have been linked to cholangiocarcinoma. Combined, the pattern of time-dependent suppression of miRNAs and the previous link to pathogenesis of hepatobiliary diseases formed a rationale for new experiments to investigate the expression behavior of those genes predicted to be targets of this subset of miRNAs at different stages of experimental biliary atresia.

**Table 1:** List of miRNAs with shared patterns of expression in EHBDs at 7 and 14 days after RRV challenge

**Common Name**	**Day 7**		**Day 14**	
	Fold change*	**P value**	Fold change*	**P Value**
mmu-miR-126-3p	−2.48	0.048	−2.84	0.041
mmu-miR-30b	−2.74	0.037	−3.22	0.049
mmu-miR-30c	−3.78	0.015	−3.25	0.013
mmu-miR-151-3p	−2.42	0.015	−2.70	0.034
mmu-miR-320	−2.36	0.019	−2.36	0.038
mmu-miR-676	−2.97	0.015	−2.12	0.026
mmu-miR-204	−3.10	0.038	−2.51	0.037
mmu-miR-193b	−2.85	0.02	−2.08	0.05
mmu-miR-365	−4.62	0.015	−4.45	0.034
mmu-miR-133a	−3.65	0.015	−2.67	0.032
mmu-miR-200b	−3.96	0.015	−2.84	0.046
mmu-miR-133b	−4.41	0.009	−4.35	0.032
mmu-miR-200a	−5.56	0.009	−3.11	0.034
mmu-miR-195	−4.93	0.014	−8.71	0.05

* Minus sign implies fold change below controls.

Fold changes are expressed in RRV-challenged mice relative to saline-injected controls.

**Table 2:** **List of previous reports of individual miRNAs in biological processes or liver diseases of the liver and bile duct based on published literature** (http://www.ncbi.nlm.nih.gov/pubmed**, as of 5/3/2012)**

**Name**	**Relationship: liver**	**Relationship: bile duct**
miR-126-3p	No report	No report
miR-30b	Inhibited TGF beta1-induced EMT, [24] downregulated after hepatectomy in rats [29]	Upregulated in LPS-induced Activated NFκB in cholangiocytes [25]
miR-30c	Required for hepatobiliary development [10]	Required for hepatobiliary development, [10] upregulated after C.parvum infection [26]
miR-151-3p	No report	No report
miR-320	Downregulated in Huh7 cells after HCV infection [21]	Downregulated in intrahepatic cholangiocarcinoma, involved in drug-triggered apoptosis [28]
miR-676	No report	No report
miR-204	No report	Downregulated in intrahepatic cholangiocarcinoma [28]
miR-193b	Downregulated in HCC, [13] upregulated in a HCC cell line after transfection of HCV genome [20]	No report
miR-365	No report	No report
miR-133a	No report	No report
miR-200b	Downregulated in HCC, [18,19] upregulated in NAFLD and NASH [22,23]	Upregulated in cholangiocarcinoma cell lines [27]
miR-133b	No report	No report
miR-200a	Downregulated in HCC [15] and inhibited the proliferation and migration of HCC cells, [17] upregulated in NAFLD [23]	No report
miR-195	Downregulated in HCC, [15] suppressed growth of HCC, [14] sensitized HCC to 5-fluorouracil, [16] inhibited proliferation of HSC [30]	No report

TGF: transforming growth factor, LPS: lipopolysaccharide, HCC: hepatocellular carcinoma, HCV: Hepatitis C virus, EMT: epithelial-to-mesenchymal transition, NAFLD: nonalcoholic fatty liver disease, NASH: nonalcoholic steatohepatitis, HSC: hepatic stellate cells.

### Increased expression of target genes in EHBDs

Because miRNAs execute their biological roles by regulating expression levels of target genes, we developed a whole-genome expression platform using RNA from EHBDs at the same time points after RRV challenge [GEO: GSE41595]. To integrate the 14 miRNAs with the expression levels of the related genes, we first built a list of putative target genes based on TargetScan and miRanda prediction algorithms, and identified 2,216 genes shared by both algorithms. Second, we mined the gene expression platform to select genes that changed by ≥2 fold either at 3, 7 and 14 days after RRV challenge when compared to saline controls, and found 1,205 genes fulfilling these criteria at P < 0.05 and 5% FDR (Additional files 5, 6 and 7). A comparison of these two lists revealed that 53 genes were shared by both lists. Third, given that miRNAs are thought to function by inhibiting the level of gene expression and that the 14 miRNAs were suppressed after RRV challenge, we examined the list of 53 genes to select genes as potential miRNA targets based on an increase in the expression by ≥2 fold in EHBDs, and got 20 genes at 7 days and 8 genes at 14 days after RRV challenge (Table 3, Additional files 8 and 9).

**Table 3:** List of potential target genes with increased expression levels in EHBDs at 7 and 14 days after RRV challenge (above saline controls)

**Day 7**		
**miRNA**	**Number of genes**	**Gene symbol**
miR-30b	8	*Pik3cd, Runx1, Ceacam1, Cysltr1, Gda, Serpine1, Socs3, Cmpk2*
miR-30c	8	*Pik3cd, Runx1, Ceacam1, Cysltr1, Gda, Serpine1, Socs3, Cmpk2*
miR-126-3p	0	
miR-133a	2	*Col8a1, Ctgf*
miR-133b	2	*Col8a1, Ctgf*
miR-151-3p	0	
miR-193b	0	
miR-195	5	*Hmga1, Il10ra, Pim1, Il7r, Has2*
miR-200a	3	*Runx1, Gbp6, Has2*
miR-200b	0	
miR-204	1	*Rnase6*
miR-320	2	*Ereg, Igf2bp3*
miR-365	1	*Arrb2*
miR-676	0	
**Day 14**		
miR-30b	6	*Ceacam1, Gda, Eya2, 1110032A04Rik, Gclc, Slc6a9*
miR-30c	6	*Ceacam1, Gda, Eya2, 1110032A04Rik, Gclc, Slc6a9*
miR-126-3p	0	
miR-133a	0	
miR-133b	0	
miR-151-3p	0	
miR-193b	0	
miR-195	1	*Tspan1*
miR-200a	2	*Fkbp5, Slc6a9*
miR-200b	0	
miR-204	0	
miR-320	0	
miR-365	0	
miR-676	0	

**Figure 2:**
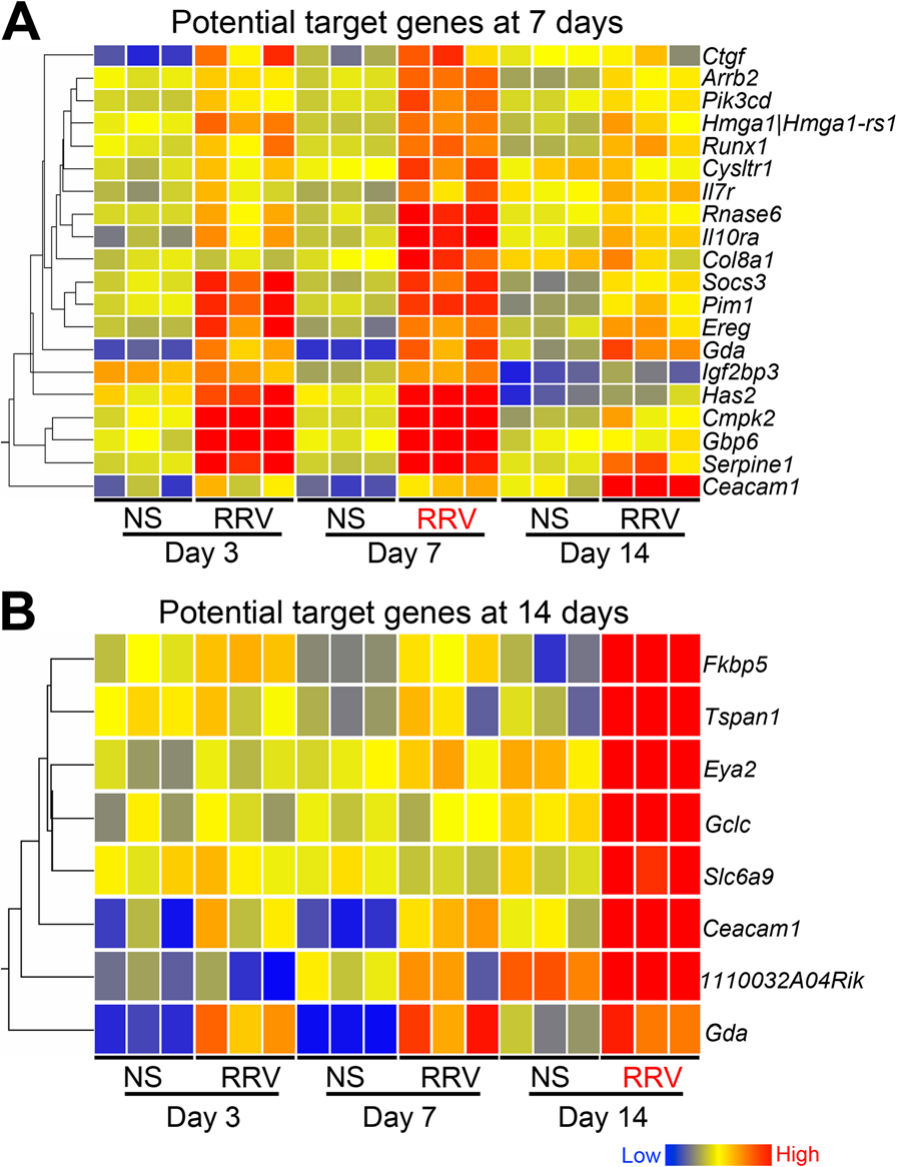
**Expression levels of potential target genes in EHBDs after RRV challenge.** One-way cluster analyses depict the changes in expression levels of mRNAs at 3, 7, and 14 days after RRV challenge relative to normal saline (NS) controls. Panel** A** shows a profile based on the differential expression at 7 days, while Panel **B** at 14 days. Each column represents expression levels from pooled samples of 2–6 EHBDs. RRV in red colored fonts indicate the time point used to select gene groups based on a significant level of expression in RRV group relative to NS controls.

Analyzing the association among the 14 miRNAs and their potential target genes (Table 3), we found that 4 miRNAs were associated with increased expression of at least 3 target genes at the time of lumenal obstruction (7 days): a) miR-30b and -30c were associated with overexpression of mRNA for *Pik3cd*, *Runx1*, *Cecam1*, *Cysltr1*, *Gda*, *Serpine1*, *Soc3* and *Cmpk2*; b) miR-195 with *Hmga1*, *Il10ra*, *Pim1*, *Il7r* and *Has2*; and c) miR-200a with *Runx1*, *Gbp6* and *Has2* (Additional file 8). Notably, although their selection was based on an overexpression at 7 days, cluster analysis showed that the increase in some of these genes began as early as 3 days and generally persisted through the time of duct atresia (Figure 2A). From the genes selected based on the increased expression at the time of atresia (14 days), 8 genes were increased, of which 6 were targets of miR-30b/c (*Ceacam1*, *Gda*, *Eya2*, *1110032A04Rik*, *Gclc*, and *Slc6a9*; Figure 2B, Table 3, Additional file 9). The linkage of coordinately overexpressed genes with a small group of miRNAs (namely miR-30b/c, -195 and 200a) formed the rationale for informatics-based analyses to explore the relationship of these genes and miRNAs in terms of biological pathways and function.

### Functional enrichment analysis of target genes

**Figure 3:**
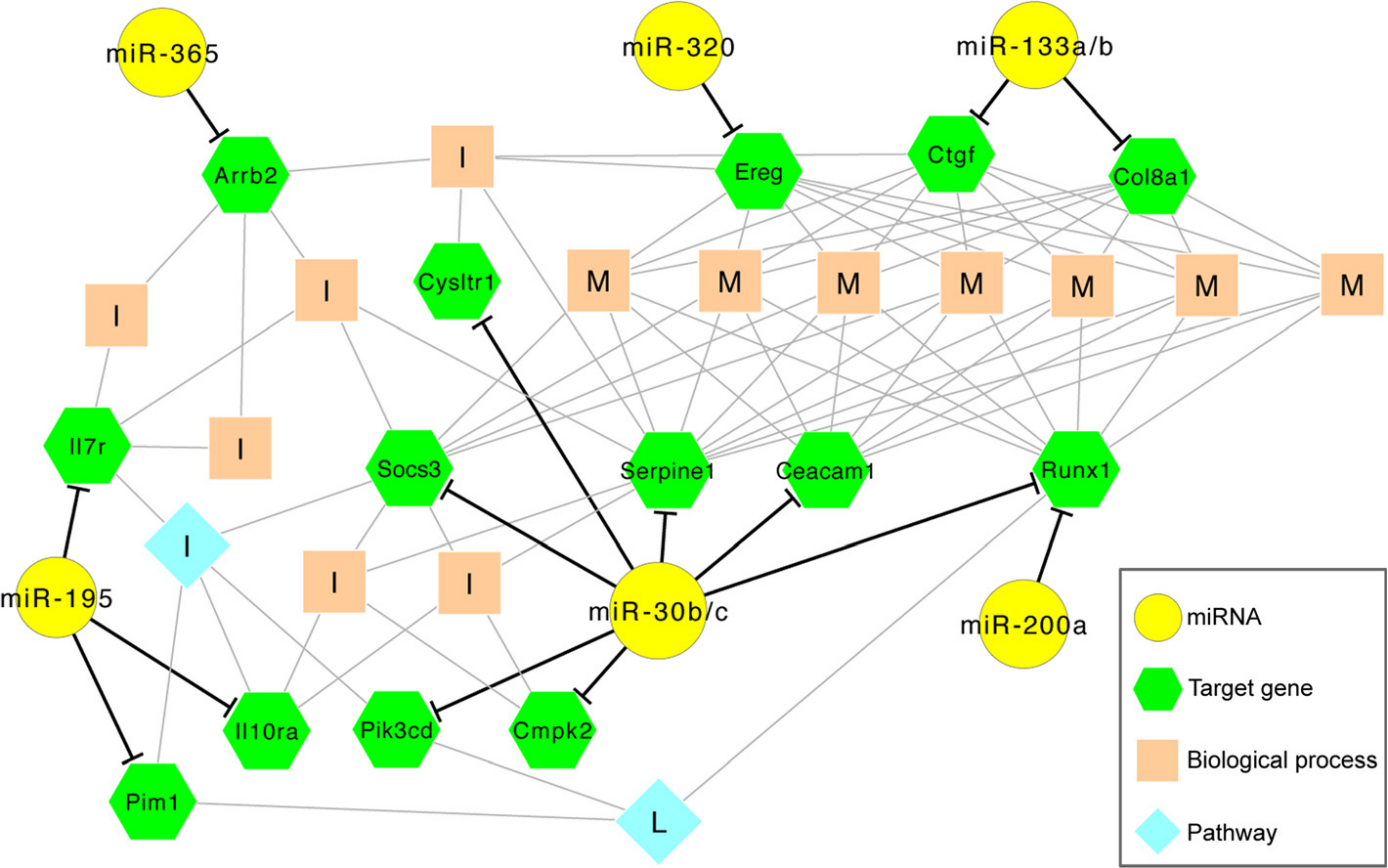
**Combined miRNA/mRNA regulatory and functional enrichment network.** The network for miRNAs and their potential targets at day 7 after RRV challenge (listed in Table 3) was drawn based on the results of functional enrichment analysis performed using ToppCluster. 13 biological processes (light brown squares) and 2 pathways (blue diamonds) were overrepresented by 8 miRNAs (yellow circles) with their 14 potential targets (green hexagons). M: Biological processes related to morphology; I: Biological processes and a pathway related to Inflammation; L: A pathway related to Leukemia.

Using ToppCluster as a platform to integrate informatics, streams of gene and protein function, regulation and animal models of disease, [31] we found 1^st^-to-3^rd^ levels of association (tier relationships) that were significantly enriched for 14 genes predicted to be targets of 8 miRNAs when compared to the entire genome (Fisher’s exact test with FDR 5%; Figure 3; Additional files 10 and 11). Among these, miR-30b/c, -195 and −365 had 1^st^ tier links to biological processes of hematology and inflammation by influencing the expression of *Pim1*, *Il10ra*, *Il7r* (miR-195), *Arrb2* (miR-365), *Pik3cd*, *Cmpk2*, *Socs3*, *Cysltr1*, *Serpine1* and *Ceacam1* (miR-30b/c). The remaining miR-200a, -320 and -133a/b were linked to organ and tissue development via *Ereg* (miR-320), *Ctgf*, *Col8a* (miR-133a/b) and *Runx1* (miR-200a and -30b/c). Thus, using integrative bioinformatics we could predict a prominent position for miR-30b/c and secondary positions for miR-133a/b, -195, -200a, -320 and −365 in a network based on the number of target genes and 1^st^ tier links with biological processes and pathways that involved the regulation of immunity and organogenesis, two classes of processes previously linked to pathogenesis of biliary atresia [2,5].

### Tissue and cellular expression of miRNAs

**Figure 4:**
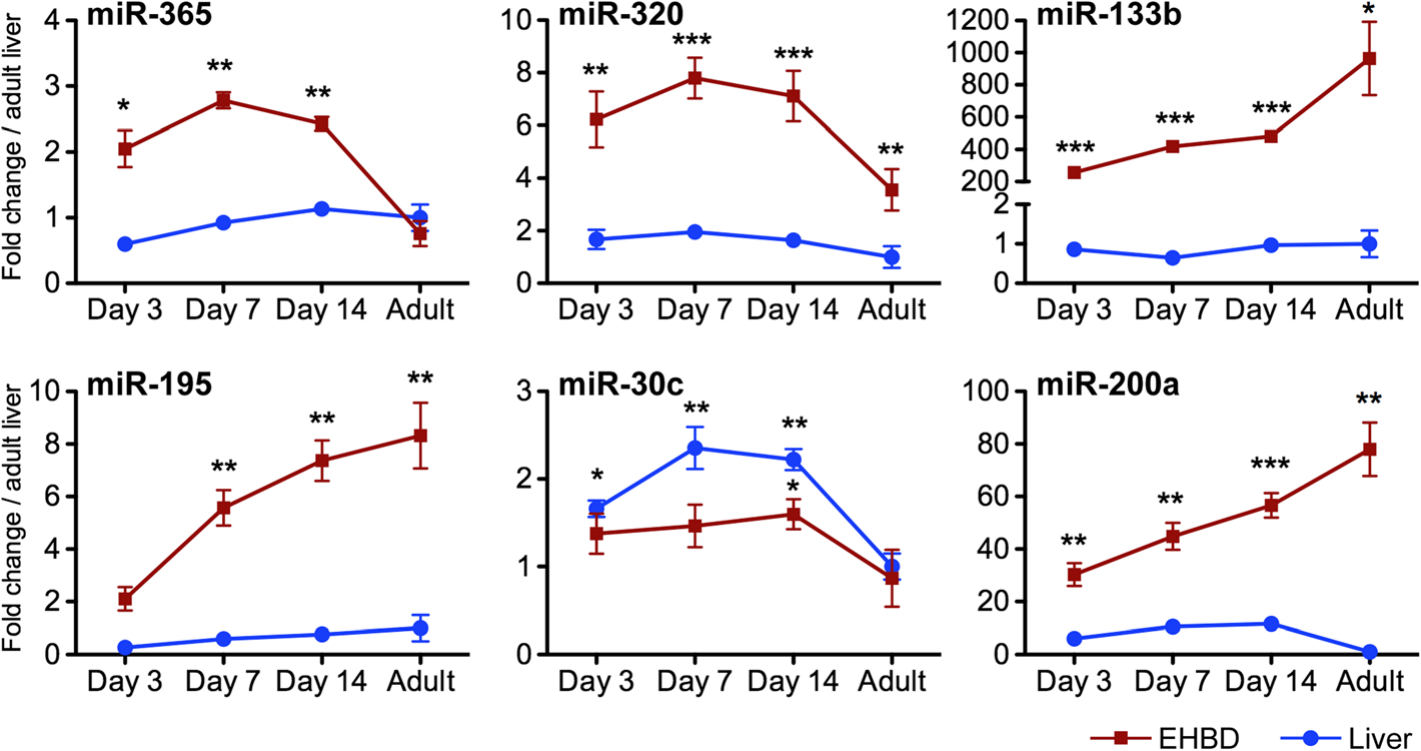
**Expression of miRNAs in the liver and extrahepatic bile ducts (EHBDs).** Expression levels in the liver and EHBDs of the same miRNAs depicted in Figure 4 during postnatal development. Expression of miRNAs was normalized against U6 miRNA, followed by fold change calculations using expression levels of adult livers. *P < 0.05, **P < 0.01, ***P < 0.001. Values are expressed as mean ± SEM.

**Figure 5:**
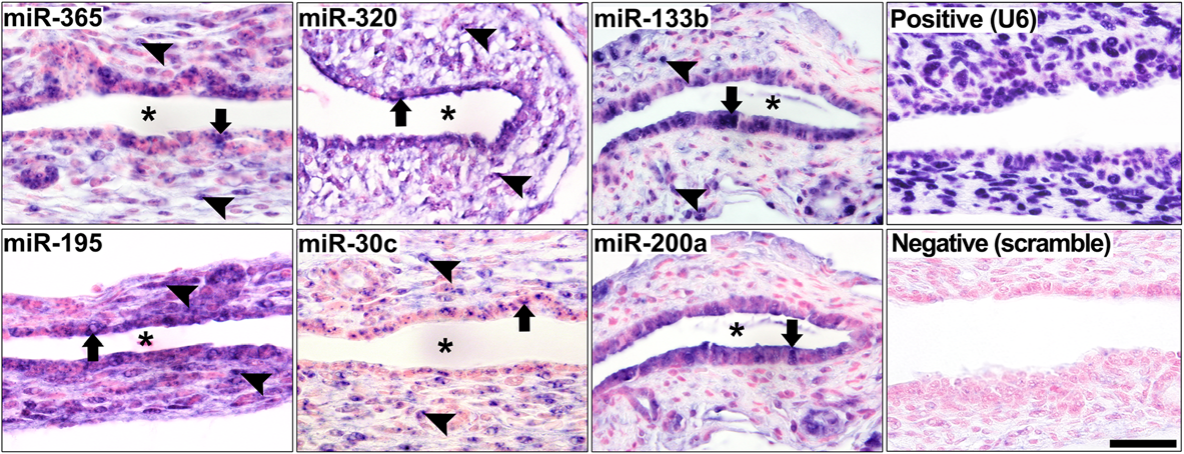
**Cellular localization of the miRNAs in EHBDs.** In situ hybridization performed on EHBDs from 7-day old neonatal mice reveals substantial expression of the miRNAs in cholangiocytes (arrows) and stromal cells in the subepithelial compartment (arrowheads). Probe for U6 microRNA served as positive control and scramble probe served as negative control. Asterisks indicate the bile duct lumen. Scale bar: 50 μm.

**Figure 6:**
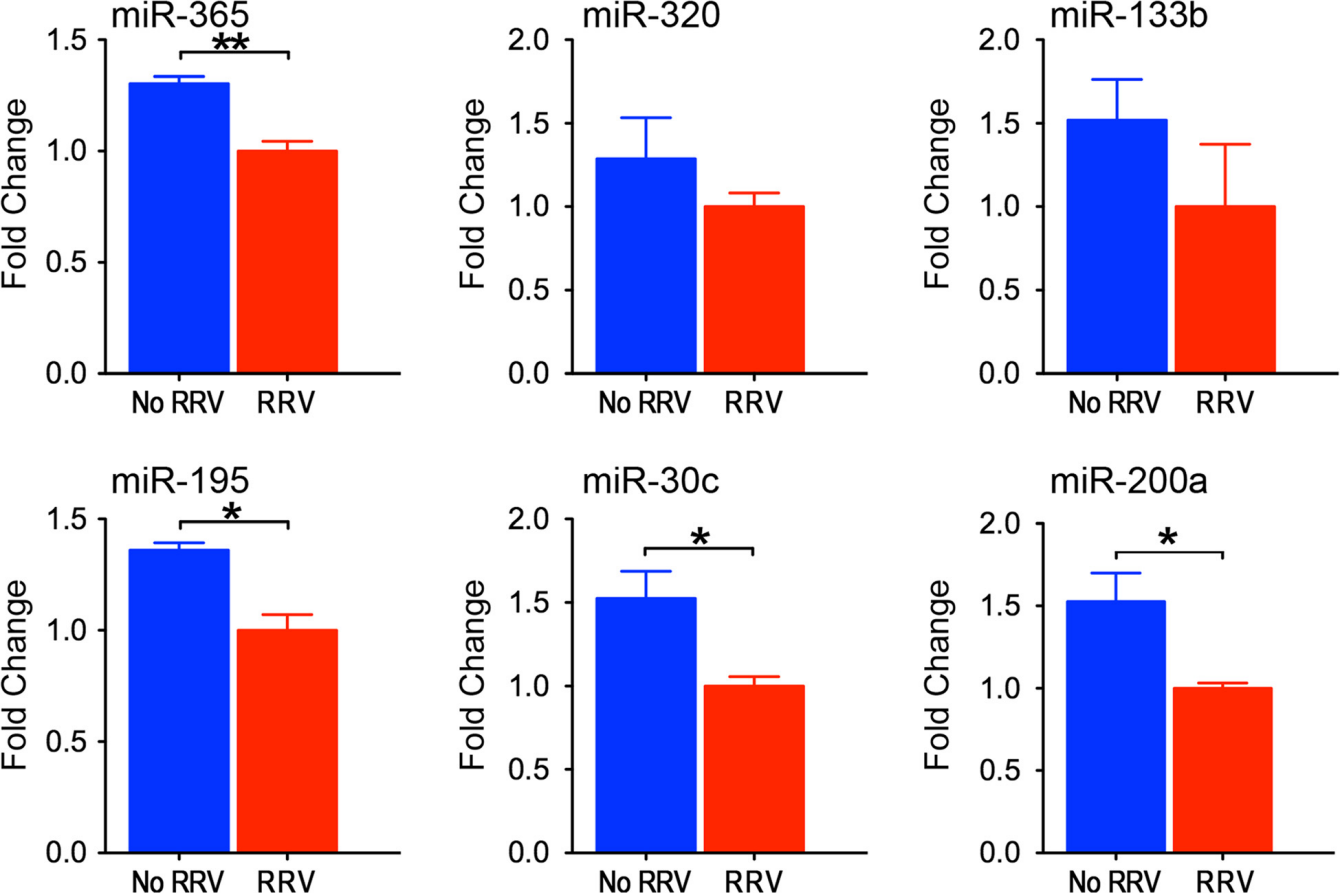
**Expression of miRNAs after RRV infection in a cultured cholangiocyte cell line.** Expression levels of the 6 miRNAs in Figure 5 were quantified 24 hours after infection with RRV at MOI of 100. Expression of miR-365, miR-195, miR-30c and miR-200a were significantly downregulated in RRV infected cholangiocytes. Expression of miRNAs was normalized against U6 miRNA, *P < 0.05, **P < 0.01. Values are expressed as mean ± SEM.

miRNAs are known to work in a cell- and tissue-specific manner and to influence hepatobiliary organogenesis [10]. Based on the period of susceptibility of Balb/c mice to RRV restricted to the first 2–3 days of life, [6,7] we determined the expression level of this select group of miRNAs in livers and EHBDs at 3, 7, and 14 days of postnatal age and 6–8 weeks of life (adults). Focusing first on the pattern of postnatal development in the liver, miR-30c and -200a increased in suckling mice at all time points by 2–12 fold above adult levels (Figure 4, blue lines), while miR-195 tended to have lower levels but did not reach statistical significance. In contrast, the developmental expression in EHBDs of miR-133b, -195 and -200a was lowest at 3 days of age and increased progressively to adult levels, while miR-365, -320 and -30c was highest during the suckling period (Figure 4, brown lines). By directly comparing the expression levels in the liver and EHBDs, we found that miR-133a/b, -195, -200a, -320 and −365 were consistently higher in EHBDs above livers at most time points, whereas miR-30c expression was lower in EHBDs when normalized to the expression level of U6 miRNA as an endogenous control (P < 0.05, Figure 4). To obtain insight into the normal expression of miRNAs at the cellular level, we performed in situ hybridization of miRNAs in EHBDs from mice at 7 days of life and found that miR-200a was expressed largely in cholangiocytes of the duct epithelium and peribiliary glands, and the remaining 5 miRNAs were expressed in cholangiocytes and subepithelial stromal cells (Figure 5). Furthermore, to investigate whether the decreased expression of the miRNAs in EHBD after RRV challenge was not a consequence of a decrease in the number of cholangiocytes during the biliary injury, we quantified the expression levels of the 6 miRNAs in a cholangiocyte cell line (mCl) cultured in the presence or absence of RRV. This in vitro system has been shown to recapitulate the cell-specific pattern of RRV infection and shared mechanism of virus-cholangiocyte interaction [8,32]. After 24 hours of RRV infection (100 multiplicity of infection [MOI]), the expression levels were downregulated by 1.30 to 1.53-fold for miR-365, miR-195, miR-30c and miR-200a (Figure 6) below levels of the vehicle control. Interestingly, the expression of miR-320 and miR-133b trended down in RRV-challenged cholangiocytes, but did not reach to significance.

## Discussion

From two comprehensive datasets displaying the expression of miRNAs and mRNAs in EHBDs at early phases of epithelial injury, onset of duct obstruction, and duct atresia, we identified miR-30b/c, -133a/b, -195, -200a, -320 and −365 as candidate miRNAs with potential roles in pathogenesis of experimental biliary atresia. The selection of miRNAs was based on a stringent statistical approach to identify miRNAs based on the highest changes in the level of expression, the simultaneous over-expression of target genes in a tissue- and time-restricted fashion, and the expression of miRNAs in the epithelial lining of EHBDs. The miRNAs formed groups with 1^st^-to-3^rd^ tier associations with genes involved in biological processes and pathways regulating tissue organogenesis and immune response, processes that have been linked to pathogenic mechanisms of biliary atresia [2,5].

miRNAs are increasingly recognized as regulators of liver physiology and diseases [11]. By regulating the levels of specific mRNAs and their posttranscriptional regulation, miRNAs have been linked to the pathogenesis of viral hepatitis, nonalcoholic fatty liver disease and steatohepatitis, fibrogenesis and hepatocellular carcinoma. For biliary atresia, a recent report identified miR-29a to be increased in the livers of neonatal mice subjected to a similar RRV infection protocol, and to target the *Igf1* and *Il1rap* genes in vivo [9]. In our study, we focused primarily on EHBDs because it is the primary site of the inflammatory response that injures cholangiocytes and produces the obstructive lesion typical of experimental atresia. Previously, we used a similar approach to survey the gene expression signature in bile ducts in search of genes or gene-groups with potential roles in pathogenesis of biliary atresia [5]. The strength of the approach was validated in subsequent studies showing that the suppression of gene groups induced by the loss of CD8+ lymphocytes [12] and natural killer [33] and dendritic cells [34] substantially decreased epithelial injury, prevented bile duct obstruction, and improved cholestasis.

Reasoning that the highest fold changes in miRNA expression in the microarray are more likely to be validated by other techniques (example: separate real-time PCR for individual miRNAs) and represent activation or suppression of their gene products, we primarily selected those miRNAs with ≥2 fold increase or decrease relative to age-matched saline-controls. This stringent approach selected 143 miRNAs, from which we focused on 14 miRNAs based on their shared expression behavior at 7 and 14 days, times when EHBDs undergo inflammatory obstruction and atresia, respectively. Among the 14 miRNAs, miR-30b/c, -133a/b, -195, -200a, -320 and −365 emerged as chief candidates of pathogenesis based on the inverse relationship with the increased expression level of their target genes in EHBD, high miRNA expression in EHBDs relative to livers (with the exception of miR-30b/c), expression in cholangiocytes of the biliary epithelium, and a decreased expression of miR-365, -195, -30 and -200a in a cholangiocyte line infected with RRV. This list of miRNAs is much smaller than the broad miRNA profile of livers from RRV-challenged mice reported by Hand et.al. [9] Notably, our findings of suppressed expression of miR-30b/c, -195, -200a and −365 in EHBDs are similar to the suppression reported previously; [9] however, the finding of high level of hepatic miR-29a was not strictly reproduced in EHBDs, which in fact had an increased expression of the family member miR-29b to 2.51 fold above saline controls at the time of duct obstruction. It is possible that different members of the miR-29 family may produce differential biological effects in a tissue-specific fashion (liver versus EHBDs), although we cannot rule out the possibility that the lower expression in our model may relate to a progressive loss of cholangiocytes populating the bile duct mucosa.

The main biological processes and diseases previously assigned to most of the 14 selected miRNAs included hepatocellular and cholangiocarcinoma, nonalcoholic fatty liver disease and steatohepatitis, and viral hepatitis infection. We also found genes associated with morphogenesis/development despite the use of control newborn mice that were age-matched to RRV-infected mice, thus avoiding the confounding variable of post-natal age. This suggests a biological scenario in which the selected genes have a role in the cellular response to RRV in addition to their traditional role in development. Perhaps more relevant to our experimental model of a biliary disease (biliary atresia), only miR-30b/c, -200b, -204 and −320 have been reported to change their expression levels in cholangiocarcinoma tissues or cell lines, [10,25-28] with miR-30 family members increasing in lipopolysaccharide-induced NFκB activation in cholangiocytes and after Cryptosporidium parvum infection, and being required for hepatobiliary development [10,25,26]. Consistent with an involvement in our model, miR-30b/c had the largest number of target genes with increased expression at the times of duct obstruction and atresia, and the largest number of 1^st^ tier links with genes regulating inflammation and immunity, which are thought to be chief biological processes involved in the pathogenesis of biliary atresia.

## Conclusion

In summary, using integrative bioinformatics to screen the biliary miRNA and mRNA expression profiles in a complementary fashion, the expression of miRNAs in a tissue and cell-specific fashion, and the predicted interaction with genes and gene-groups, we identified miR-30b/c, -133a/b, -195, -200a, -320 and −365 as candidate miRNAs with potential roles in pathogenesis of experimental biliary atresia. Among these, our data point to miR-30b/c as an attractive miRNA for future mechanistic studies to define how its expression in cholangiocytes and subepithelial cells modulate pathogenic mechanisms of disease by regulation of their target genes.

## Methods

### Mouse model of experimental biliary atresia

Balb/c mice were purchased from Charles River Laboratories (Wilmington, MA). Newborn mice were injected with 20 μL of a solution containing 1.5×10^6^ fluorescent-forming units (ffu) of RRV or 0.9% NaCl (saline) intraperitoneally within 24 hours of birth, as described previously [5,8]. The extrahepatic bile ducts and gallbladder were microdissected en bloc (here collectively referred to as EHBDs) at 3, 7 and 14 days after RRV or saline injection and stabilized using RNAlater (QIAGEN, CA) or embedded in paraffin. For RNA extraction, 3 sets were generated for each time point and experimental and control groups, with each set containing 2–6 EHBDs depending on their sizes to make sure enough RNA was available for use in experiments quantifying the expression of miRNA and mRNA. All mice received appropriate care consistent with criteria outlined in the “Guide for the Care and Use of Laboratory Animals” prepared by the National Academy of Sciences and published by the National Institutes of Health. The Institutional Animal Care and Use Committee of the Cincinnati Children’s Research Foundation approved all animal protocols.

### miRNA expression arrays

To quantify miRNA expression, we first isolated total RNA from EHBDs of RRV- or saline-injected mice using the miRNeasy Mini Kit, according to the manufacturer’s protocol (QIAGEN); RNA integrity was verified using the Agilent 2100 Bioanalyser (Agilent Technologies, Inc., CA). Then, reverse transcription into first strand cDNA was performed using reagents from TaqMan® MicroRNA RT Kit and Megaplex RT Primer Pools (Applied Biosystems, CA). Quantitative real-time PCR was performed using the TaqMan® Array Rodent MicroRNA Card v2.0 (A and B) with the Universal PCR Master Mix in 7900 HT Fast Real-Time PCR system (Applied Biosystems). All samples were run in triplicate and the relative expression was calculated using the comparative Ct method, [35] in which average Ct values of four MammU6 probes on each plate were used as reference. The ΔCt values were imported into GeneSpring GX 11.5 platform (Agilent Technologies) for statistical analyses.

### mRNA expression arrays

Aliquots from total RNA from similar groups of EHBDs were also applied to a genome-wide expression protocol consisting of the synthesis of biotinylated cRNAs from 400 ng aliquots. cRNA pools were hybridized to the oligonucleotide-based GeneChip® Mouse Gene 1.0 ST Array (Affymetrix, CA), which contains probe sets to quantify 28,853 genes, scanned, and monitored for specific signals with the GeneChip® Operating Software, as described previously [5]. Affymetrix CEL files were imported into GeneSpring GX11.5 platform and further analyzed.

### miRNA target prediction and enrichment analyses

Putative targets of the miRNAs were identified using Target Scan (http://www.targetscan.org) [36] and miRanda (http://www.microrna.org/microrna/home.do), mouse target site predictions with good mirSVR score and conserved miRNA, August 2010) [37]. The genes that were listed in both prediction algorithms were adopted as putative target genes for individual miRNAs. For functional enrichment analysis, we used ToppCluster (http://toppcluster.cchmc.org) [31] with Fisher’s exact test and false discovery rate (FDR) correction to control for over-representation of biological contexts. Combined miRNA/mRNA regulatory and functional enrichment network was drawn using Cytoscape [38].

### Quantitative real-time PCR

For analyses of developmental regulation of specific miRNAs, RNA pools from livers and EHBDs harvested from healthy newborn Balb/c mice at days 3, 7 and 14 of life and from 6–8 week Balb/c adult mice underwent quantitative reverse-transcription PCR using TaqMan® Universal PCR Master Mix and specific miRNA primers (Applied Biosystems).

### In situ hybridization

Formalin-fixed paraffin-embedded EHBDs from 7-day old Balb/c mice were subjected to in situ hybridization, using miRCURY LNA^TM^ microRNA Detection Probes (Exiqon, Vedbaek, Denmark) and an automated Discovery XT system (Ventana Medical Systems, AZ), according to manufacturer’s protocol. Rabbit anti-digoxin antibody (Sigma-Aldrich, MO) and ChromoMab Blue kit (Ventana Medical Systems) were used to colorize the signals, and the slides were counterstained with Nuclear Fast Red (Polyscientific, NY).

### In vitro model of experimental biliary atresia

To quantify miRNA expression in cholangiocytes infected with RRV, an immortalized cholangiocyte cell line (mCl) derived from BALB/c mice was infected with RRV as described previously [32]. In brief, mCl was cultured in 24-well culture plate and grown to 90-95% confluence. After counting cell numbers in one well per plate, cells in the remaining wells were washed with Earl’s Balanced Salt Solution and overlaid with 200 μl of serum-free DMEM and 4 μl/mL trypsin containing live virus at MOI = 100; the same solution without virus served as control. After 24 hours of incubation, the cells were washed once with phosphate buffered saline and total RNA was isolated using the miRNeasy Mini Kit, according to the manufacturer’s protocol (QIAGEN). Then, quantitative reverse-transcription PCR was performed using TaqMan® Universal PCR Master Mix and specific miRNA primers (Applied Biosystems). All samples were run in duplicate and the relative expression was calculated using MammU6 as reference.

### Statistical analysis

Conventional statistical procedures were applied for miRNA and mRNA gene array experiments using GeneSpring GX11.5 platform (t-test with a significance of 0.05 and Benjamini-Hochberg multiple testing correction (FDR 0.05). Fisher’s exact test was applied to determine strength of association between miRNAs and gene functions. For comparative analyses of real-time PCR data among developmental groups, we used t-test with statistical significance set at P < 0.05.

### Availability of supporting data

The dataset supporting the results of this article is available in the Gene Expression Omnibus repository (http://www.ncbi.nlm.nih.gov/geo/), with accession code GSE41595.

## Abbreviations

RRV: Rhesus rotavirus; EHBDs: Extrahepatic bile ducts; FDR: False discovery rate.

## Competing interests

The authors report no conflict of interest.

## Authors’ contribution

KB and KS conducted all murine experiments and data analysis. RS, PS, RM and SW performed microarray experiments, validation of miRNA and mRNA expression levels, and in situ hybridization. VK, ED and AGJ conducted and oversaw analyses of expression platforms. JAB oversaw study design, data analysis, and validation experiments. All authors contributed to the draft of the manuscript. All authors read and approved the final manuscript.

## Supplementary Material

Additional file 1 miRNAs differentially expressed in extrahepatic bile ducts at 3, 7 and 14 days after RRV challenge when compared to saline controls (≥2 fold change, 5% FDR). Click here for file

Additional file 2 **Differential expression of miRNAs in EHBDs after RRV challenge.** Numbers of miRNAs differentially expressed in EHBDs by at least 2 fold at 3, 7 and 14 days after RRV challenge relative to saline controls. Click here for file

Additional file 3 **Expression profile of miRNAs in EHBD.** One-way cluster analysis depicts the expression levels of 143 miRNAs differentially expressed in EHBDs after RRV challenge relative to normal saline (NS) controls. Each column represents expression levels from pooled samples of 2–6 EHBDs. The miRNAs in red font were suppressed on both days 7 and 14. Click here for file

Additional file 4 **Expression levels of 2 family members of miR-29 on day 7 after RRV challenge are shown as fold change to saline condition.** In this study, miR-29a was not regulated in EHBD, but miR-29b was upregulated by 2.51-fold on day 7 after RRV challenge. Values are expressed as mean ± SEM. *P < 0.05. Click here for file

Additional file 5 **Differential expression of mRNAs in EHBDs after RRV challenge.** Numbers of mRNAs differentially expressed in EHBDs by at least 2 fold at 3, 7 and 14 days after RRV challenge relative to saline controls. Click here for file

Additional file 6 List of genes that changed the levels of expression by ≥2 fold after RRV on day 7 (when compared to saline controls; P < 0.05 and 5% FDR). Click here for file

Additional file 7 List of genes that changed the levels of expression by ≥2 fold after RRV on day 14 (when compared to saline controls; P < 0.05 and 5% FDR). Click here for file

Additional file 8 **Expression levels of miRNAs and their potential target genes on day 7 (shown in Table **3**), shown as fold change to RRV for miRNAs and to saline controls for mRNAs.** Note that all miRNAs were downregulated and all mRNAs were upregulated by more than 2-fold in the RRV group. Values are expressed as mean ± SEM. Click here for file

Additional file 9 **Expression levels of 4 miRNAs and their potential target genes on day 14 (shown in Table **3**, shown as fold change to RRV for miRNAs and to saline controls for mRNAs.** Note that all miRNAs were downregulated and all mRNAs were upregulated by more than 2-fold in RRV condition. Values are expressed as mean ± SEM. Click here for file

Additional file 10 List of categories that were overrepresented by the 20 potential target genes on day 7. Click here for file

Additional file 11 **Details of components of the miRNA/mRNA regulatory and functional enrichment network depicted in Figure** 3**.** The network for miRNAs and their potential targets at day 7 (listed in Table 3) was drawn based on the results of functional enrichment analysis performed using ToppCluster. 13 biological processes (light brown squares) and 2 pathways (blue diamonds) were overrepresented by 8 miRNAs (yellow circles) with their 14 potential targets (green hexagons). Click here for file
