# Supplementary figures and images for: Integrative genomics identifies candidate microRNAs for pathogenesis of experimental biliary atresia
Source: BMC Syst Biol. 2013 Oct 20;7:104. doi: 10.1186/1752-0509-7-104 (PMC3819657; doi:10.1186/1752-0509-7-104)

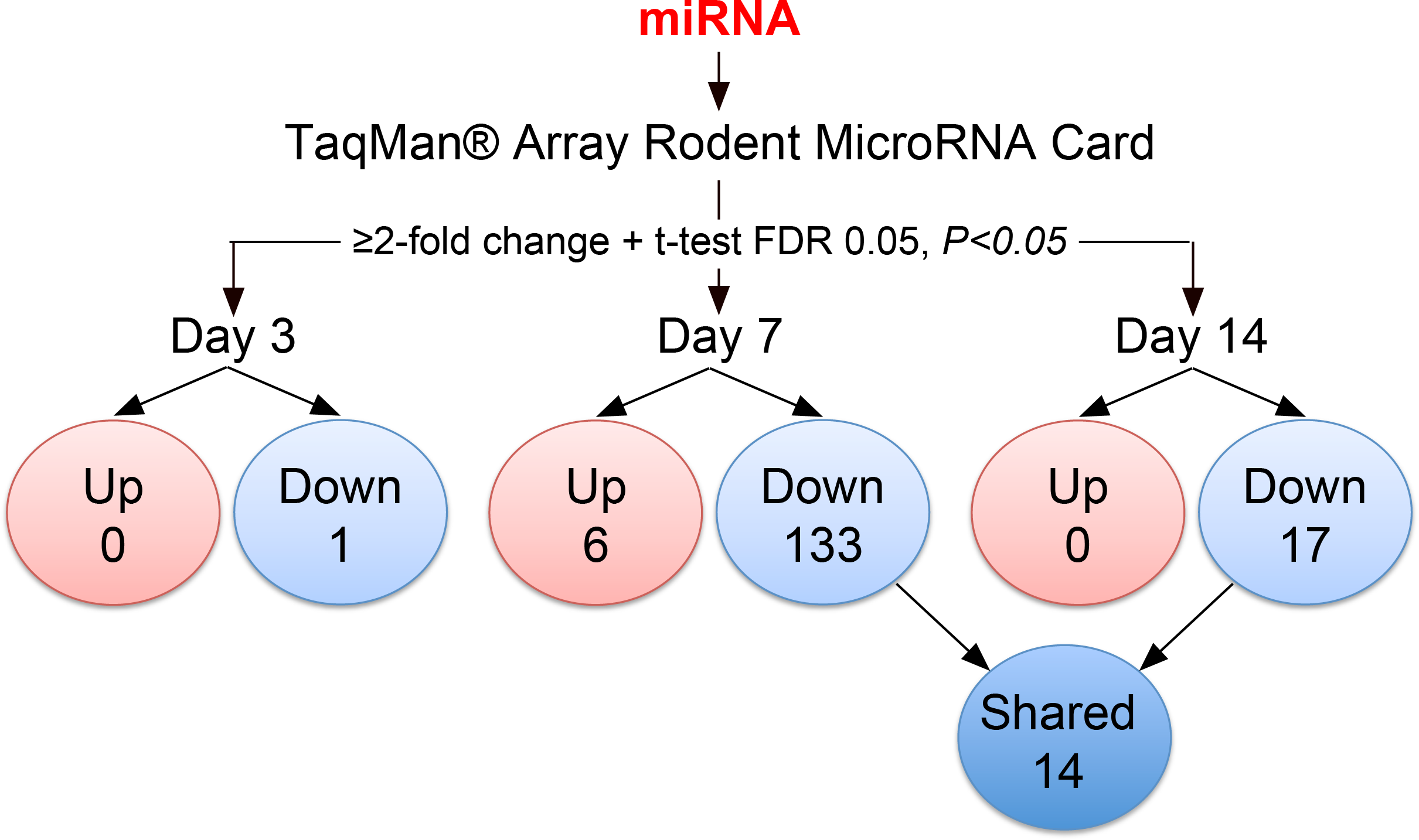

Supplement: Additional file 2 — Differential expression of miRNAs in EHBDs after RRV challenge. Numbers of miRNAs differentially expressed in EHBDs by at least 2 fold at 3, 7 and 14 days after RRV challenge relative to saline controls. [file 1752-0509-7-104-S2.png]

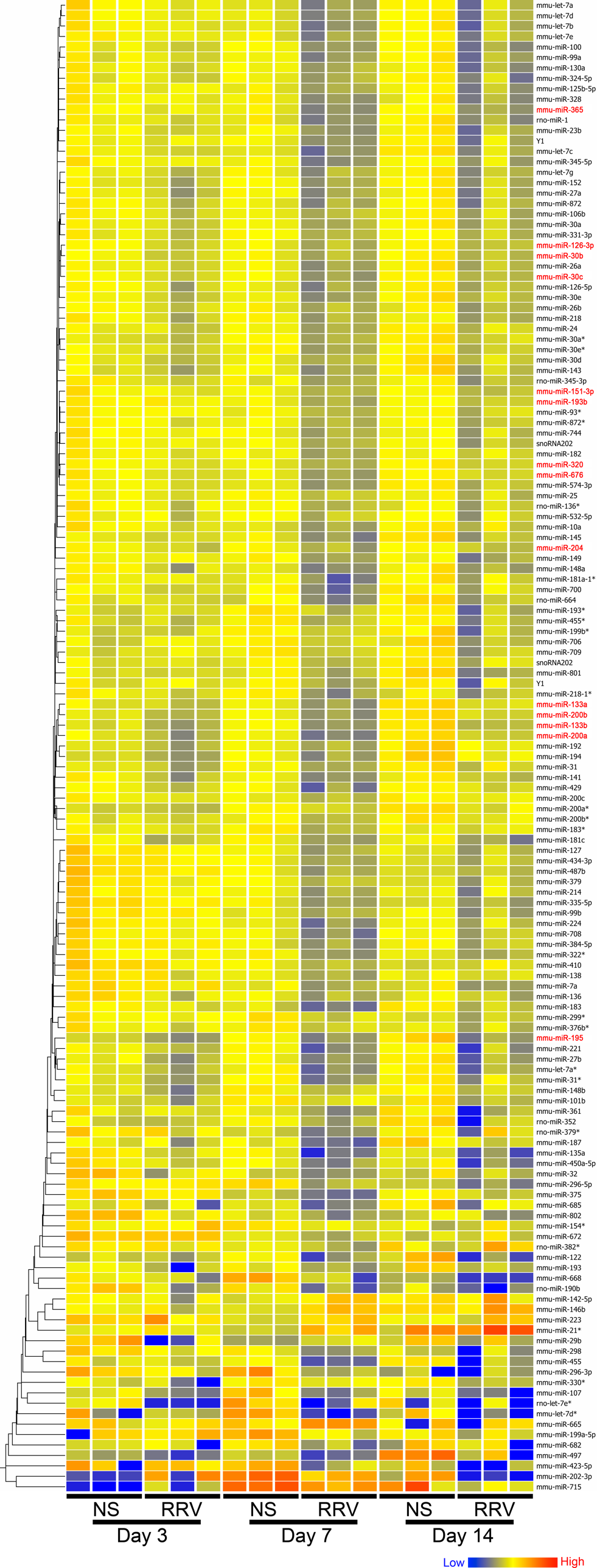

Supplement: Additional file 3 — Expression profile of miRNAs in EHBD. One-way cluster analysis depicts the expression levels of 143 miRNAs differentially expressed in EHBDs after RRV challenge relative to normal saline (NS) controls. Each column represents expression levels from pooled samples of 2–6 EHBDs. The miRNAs in red font were suppressed on both days 7 and 14. [file 1752-0509-7-104-S3.png]

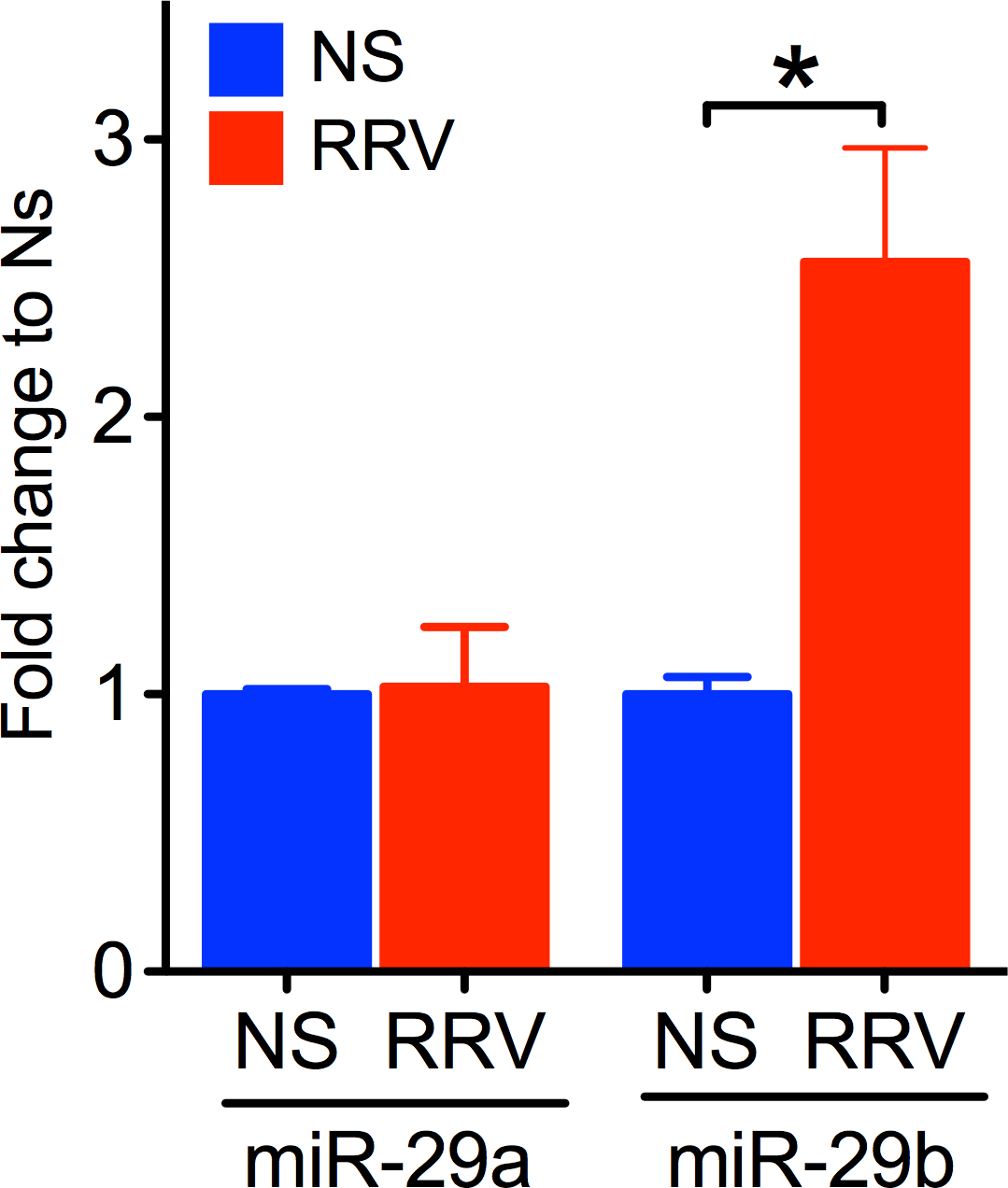

Supplement: Additional file 4 — Expression levels of 2 family members of miR-29 on day 7 after RRV challenge are shown as fold change to saline condition. In this study, miR-29a was not regulated in EHBD, but miR-29b was upregulated by 2.51-fold on day 7 after RRV challenge. Values are expressed as mean ± SEM. *P < 0.05. [file 1752-0509-7-104-S4.png]

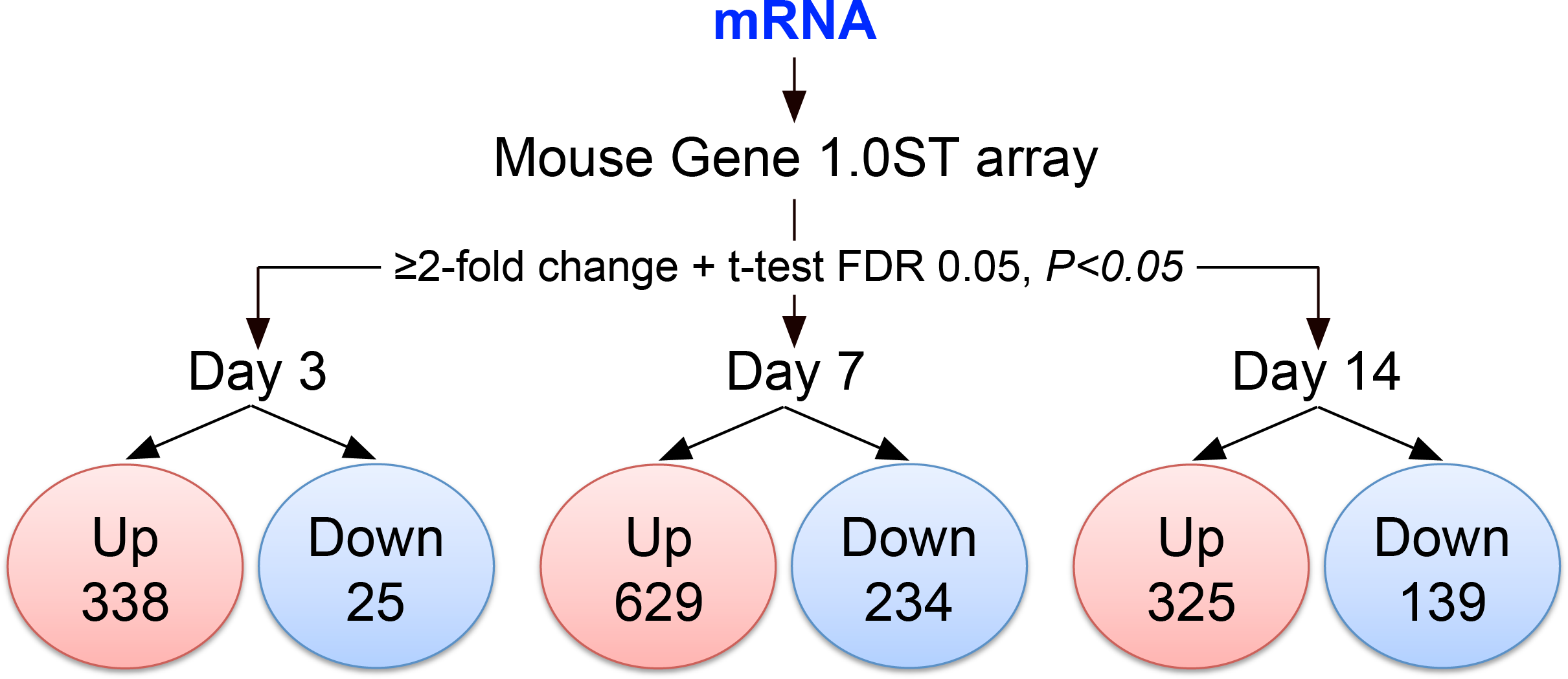

Supplement: Additional file 5 — Differential expression of mRNAs in EHBDs after RRV challenge. Numbers of mRNAs differentially expressed in EHBDs by at least 2 fold at 3, 7 and 14 days after RRV challenge relative to saline controls. [file 1752-0509-7-104-S5.png]

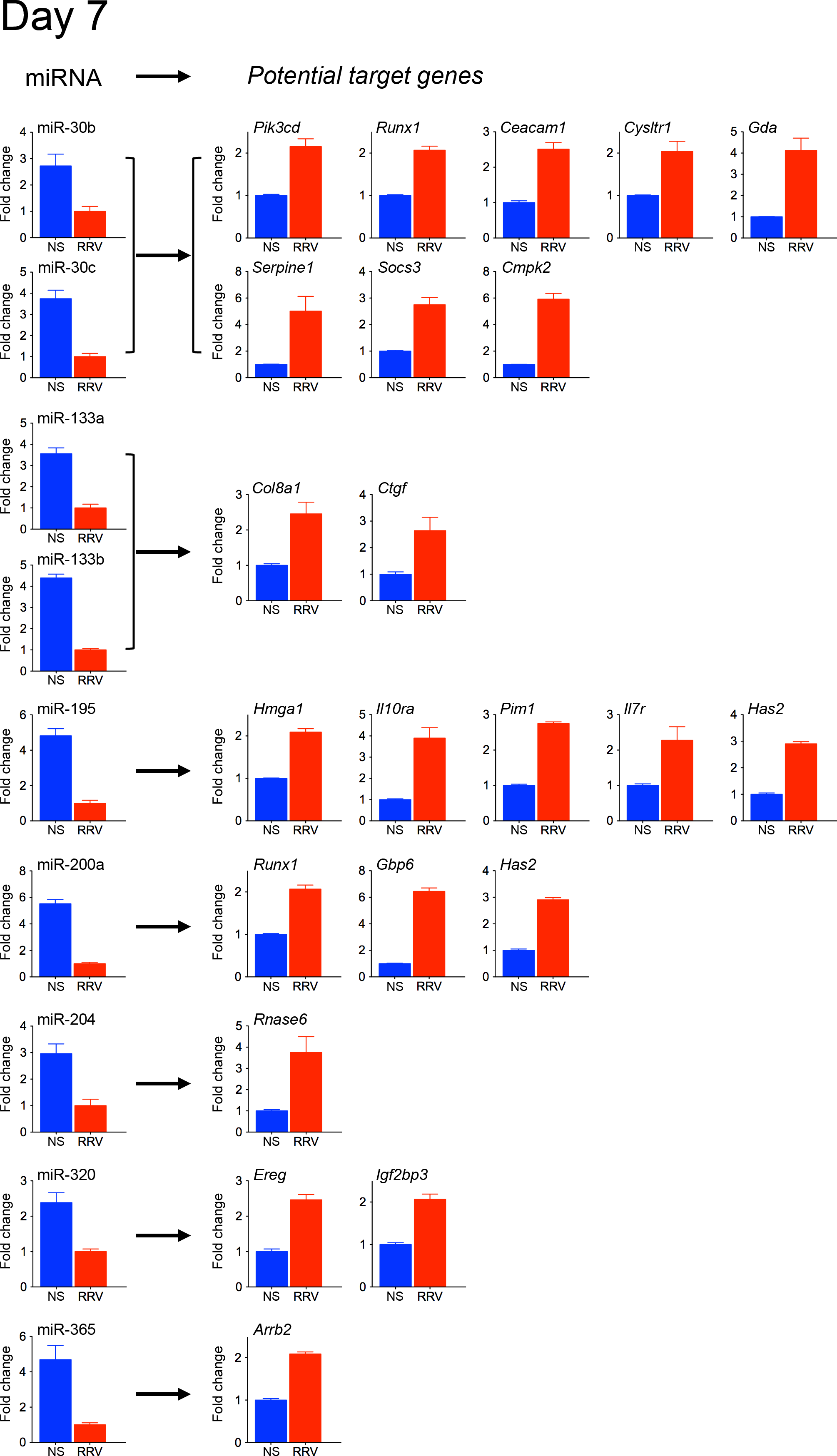

Supplement: Additional file 8 — Expression levels of miRNAs and their potential target genes on day 7 (shown in Table 3), shown as fold change to RRV for miRNAs and to saline controls for mRNAs. Note that all miRNAs were downregulated and all mRNAs were upregulated by more than 2-fold in the RRV group. Values are expressed as mean ± SEM. [file 1752-0509-7-104-S8.png]

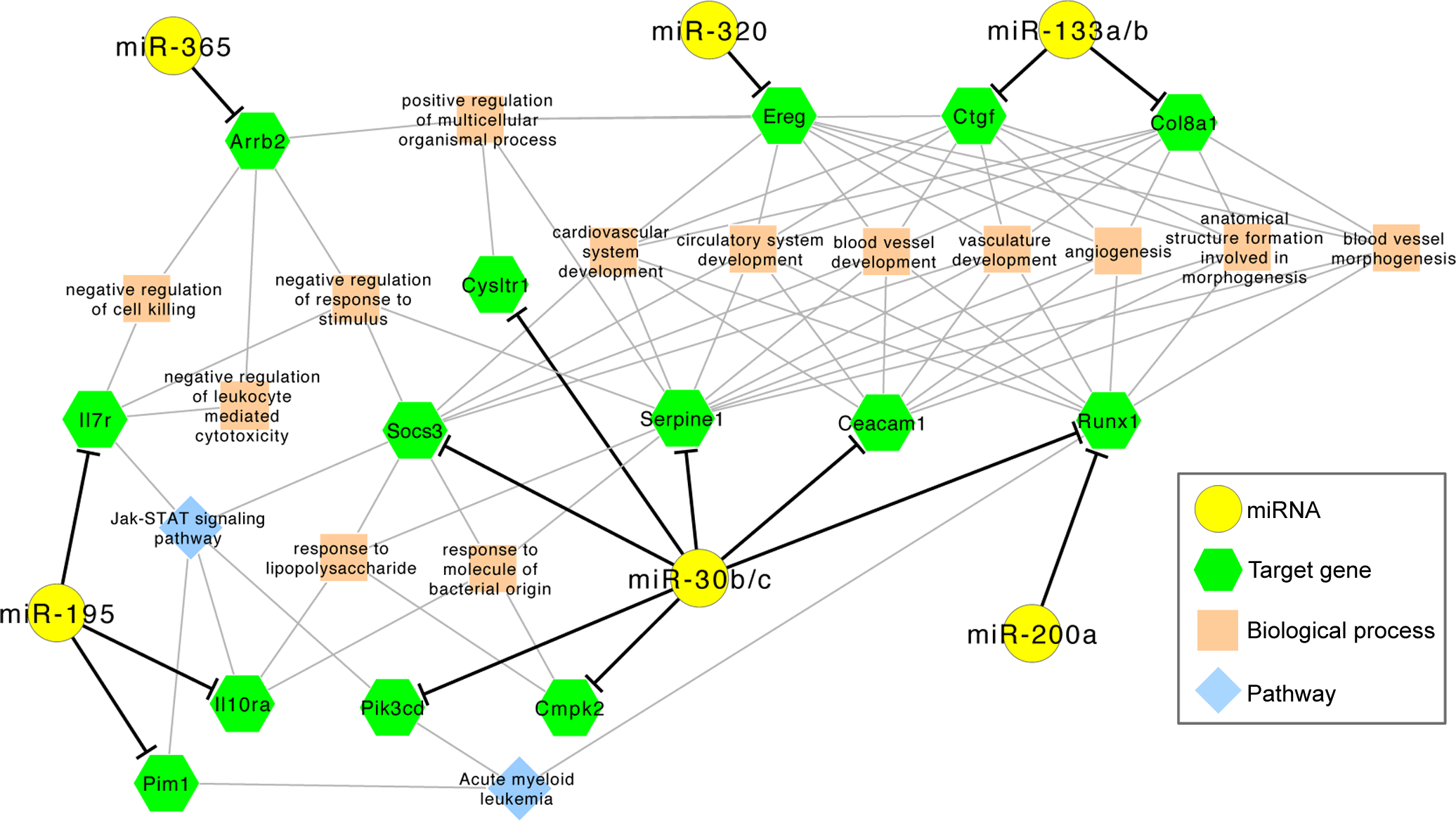

Supplement: Additional file 11 — Details of components of the miRNA/mRNA regulatory and functional enrichment network depicted in Figure 3. The network for miRNAs and their potential targets at day 7 (listed in Table 3) was drawn based on the results of functional enrichment analysis performed using ToppCluster. 13 biological processes (light brown squares) and 2 pathways (blue diamonds) were overrepresented by 8 miRNAs (yellow circles) with their 14 potential targets (green hexagons). [file 1752-0509-7-104-S11.png]
